# A case report and follow-up of the first live birth after heterotopic transplantation of cryopreserved ovarian tissue in Eastern Europe

**DOI:** 10.1186/s12905-019-0764-8

**Published:** 2019-05-14

**Authors:** Triin Tammiste, Keiu Kask, Peeter Padrik, Külli Idla, Karin Rosenstein, Tatjana Jatsenko, Piret Veerus, Andres Salumets

**Affiliations:** 1 West Tallinn Central Hospital Women’s Clinic, Tallinn, Estonia; 2 Internal Medicine Clinic, Institute of Clinical Medicine, University of Tartu, Tartu, Estonia; 3 Women’s Clinic, Institute of Clinical Medicine, University of Tartu, Tartu, Estonia; 4 Cancer Centre of Tartu University Hospital, Tartu, Estonia; 5 Department of Hematology and Oncology, Institute of Clinical Medicine, University of Tartu, Tartu, Estonia; 6 Nova Vita Clinic, Tallinn, Estonia; 7 Competence Centre on Health Technologies, Tartu, Estonia; 8 Institute of Biomedicine and Translational Medicine, University of Tartu, Tartu, Estonia; 9 Department of Obstetrics and Gynecology, University of Helsinki and Helsinki University Hospital, Helsinki, Finland

**Keywords:** Fertility preservation, Ovarian tissue cryopreservation, Heterotopic transplantation, Restoration of gonadal function, Live birth, Case report

## Abstract

**Background:**

Ovarian insufficiency is a major concern for long-term cancer survivors. Although semen freezing is well established to preserve male fertility, the possibilities to secure post-cancer female fertility are mostly limited to oocyte or embryo freezing. These methods require time-consuming ovarian stimulation with or without in vitro fertilization (IVF) that evidently delays cancer therapy. Ovarian tissue cryopreservation and subsequent thawed tissue autotransplantation are considered the most promising alternative strategy for restoring the fertility of oncology patients, which has not yet received the full clinical acceptance. Therefore, all successful cases are needed to prove its reliability and safety.

**Case presentation:**

Here we report a single case in Estonia, where a 28-year-old woman with malignant breast neoplasm had ovarian cortex cryopreserved before commencing gonadotoxic chemo- and radiotherapy. Two years after cancer therapy, the patient underwent heterotopic ovarian tissue transplantation into the lateral pelvic wall. The folliculogenesis was stimulated in the transplanted tissue by exogenous follicle-stimulating hormone and oocytes were collected under ultrasound guidance for IVF and embryo transfer. The healthy boy was born after full-term gestation in 2014, first in Eastern Europe.

**Conclusion:**

Despite many countries have reported the first implementation of the ovarian tissue freezing and transplantation protocols, the data is still limited on the effectiveness of heterotopic ovarian transplant techniques. Thus, all case reports of heterotopic ovarian tissue transplantation and long-term follow-ups to describe the children’s health are valuable source of clinical experience.

## Background

Women are born with roughly one million follicle-enclosed oocytes, with the vast majority of them undergoing atresia and only 300–400 fully matured eggs becoming available for fertilization during the entire woman’s reproductive life-span. In addition, the ovaries are very sensitive to different medical treatments, particularly cytotoxic treatments of oncotherapies, such as using alkylating agents and ionizing radiation, rendering women with cancer prematurely sterile due to the iatrogenic manipulations [1, 2]. The improvements in cancer treatments over the last decades have resulted in vastly increased numbers of long-term survivors, while the loss of fertility remains as one of the major life-quality concerns for young women facing potentially sterilising treatment [1, 3].

Several options are available to preserve fertility in cancer patients, giving them the opportunity for motherhood when they successfully overcome their disease: immature or mature oocyte cryopreservation; embryo freezing and ovarian tissue cryopreservation [4]. Only the latter is suitable for pre-pubertal girls with immature eggs only and women who cannot delay the start of chemotherapy and radiation for cancer therapy [4, 5]. Despite the preliminary results are encouraging as orthotopic reimplantation of cryopreserved ovarian tissue restores ovarian activity in > 95% of women [6], the location and timing of the transplantation are not fully studied and unequivocally agreed in clinical guidelines.

Success rate after ovarian tissue reimplantation is steadily increasing and the live birth rate is estimated to be around 35–40% [6, 7]. The first human live birth as a result of ovarian cortex reimplantation was reported in 2004 [8], and since then there are > 100 live births reported globally by multicenter and single case reports [6, 9]. However, the global access to this procedure remains poor, which also impedes the evaluation of the overall success and safety of the method.

Here, we report the first case in Estonia, where a 28-year-old woman with malignant neoplasm of breast tissue had ovarian cortex cryopreserved for fertility preservation. The subsequent heterotopic transplantation, stimulated folliculogenesis, IVF and embryo transfer resulted in a successful pregnancy and a healthy child born in 2014, being the first live birth after ovarian tissue transplantation in Eastern Europe. We also report a spontaneous pregnancy and second live birth in 2017 to the same patient, demonstrating that the personalised prediction for fertility restoration is still hard to make. The health follow-up for both children is also provided, contributing to the emerging understanding of the overall safety of the procedure.

## Case presentation

### Patient information

**Fig. 1:**
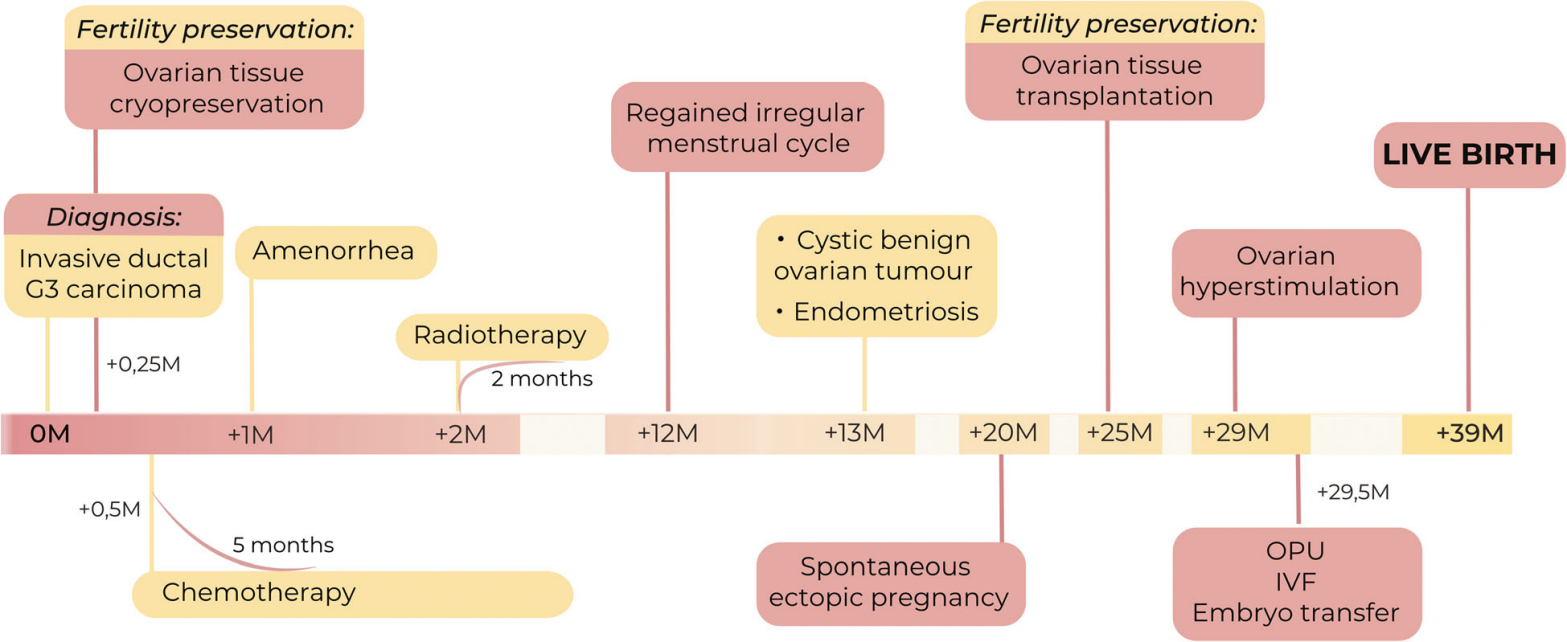
Timescale of the patient’s medical history from the diagnosis of the cancer to the successful live birth. A 28-year-old woman with invasive ductal G3 carcinoma had ovarian cortex cryopreserved before commencing gonadotoxic chemo- and radiotherapy. Two years after cancer therapy, the patient underwent heterotopic ovarian tissue transplantation into the lateral pelvic wall. The folliculogenesis was stimulated in the transplanted tissue by exogenous follicle-stimulating hormone and oocytes were collected under ultrasound guidance for IVF and embryo transfer. The healthy boy was born after full-term gestation, first in Eastern Europe

We report a case of the patient, a 28-year-old woman with a regular 28-day menstrual cycle (FSH level 5,1 U/L), who was diagnosed with carcinoma of the left breast. The overall timeline of the medical procedures from cancer diagnosis to the birth of a child is shown in Fig. 1.

In cancer diagnosis, the postoperative pathology revealed poorly differentiated invasive ductal high-grade carcinoma, with negative sentinel node biopsy (pT2 pN0 M0 G3 stage IIA by TNM classification) in 2010. The patient underwent targeted sequencing of cancer tissue using TruSight Cancer panel (Illumina, Cat No FC-121-0202) of mutational hotpots in genes predisposing or associated with cancer. Analysis of the targeted regions did not reveal any cancer-related variants/mutations in *BRCA1, BRCA2, PTEN, STK11, CDH1, CHEK2, BRIP1, ATM, PALB2, NF1* and *TP53* genes.

### Ovarian tissue cryopreservation

The option of ovarian tissue cryopreservation was offered as fertility preservation because of the time limitation before chemotherapy, as there was not enough time for hormonal stimulation to produce mature oocytes for freezing. It was also known that the cancer therapy planned for the patient (chemotherapy and radiotherapy) would be highly gonadotoxic and would likely lead to premature ovarian insufficiency and a loss of menstrual cycle activity. The patient granted her written informed consent for conducting the procedure.

During laparoscopy, two ovarian pieces, sized 1.5 × 1.0 cm, were obtained from both sides, containing ovarian cortex and medullar part. The ovarian cortex was manually dissected from medullary tissue in HEPES-buffered IVF culture media (Origio, Denmark). The cortical part of the tissue was cut into smaller pieces (1-2 mm × 3-5 mm) with a thickness of 1-2 mm. For cryopreservation, the cortical slices were incubated with human serum albumin (HSA, 25 mg/ml) for 5 min, 1.5 M 1,2-propanediol (PrOH) for 10 min, and 1.5 M PrOH and 0.1 M sucrose for 5 min in PBS (Sigma-Aldrich, Germany) at room temperature; were transferred to cryovials and were frozen using slow freezing protocol as described previously [10].

### Adjuvant cancer therapy

After the surgery for breast cancer and laparoscopy to remove the ovarian tissue, the patient was eligible for adjuvant chemotherapy and received six cycles of the FEC regimen containing a sequential combination of anthracyclines: three cycles of 5-fluorouracil 500 mg/m^2^ (800mg), epirubicin 80 mg/m^2^ (130mg) and cyclophosphamide 500 mg/m^2^ (dosage 800 mg intravenous). Since the patient developed neutropenia during the chemotherapy, reduced doses of anthracyclines were administered for the last three cycles: 5-fluorouracil (600 mg), epirubicin (110 mg) and cyclophosphamide (dosage 600 mg). The chemotherapy lasted for 5 months in 2010–2011, followed by radiotherapy to the breast tissue (50Gy) and boost to the operation scar (10Gy) for two months in 2011. The patient developed amenorrhea shortly after the initiation of chemotherapy. However, no hormonal tests are available to the authors as they were made in another hospital.

### Ovarian tissue transplantation

After chemo- and radiotherapy-induced amenorrhea, the patient regained rare spontaneous menstrual cycles at the beginning of 2012. Her FSH level was 22,1 U/L and AMH level was 0,04 ng/mL. Despite of the irregular oligomenorrhea, a spontaneous ectopic pregnancy was diagnosed in the right fallopian tube in the same year. After removing the ectopic pregnancy, the irregular oligomenorrhea lasted until 2013, indicating the deterioration of the ovarian follicular pool after cancer treatment. This was considered as a strong indication for using the ovarian tissue grafting to obtain the pregnancy, when the patient was subsequently counselled for her wish for motherhood.

The decision to use the heterotopic ovarian transplantation was based on the patient’s clinical conditions, what prevented the ovarian tissue orthotopic transplantation. Briefly, recurrent laparoscopies had revealed several pelvic pathologies: before and after cancer therapy, recurrent cystic benign ovarian tumours were found, arising from the left ovary. The tumour completely filled the rectouterine pouch (cavum Douglas) and dislocated the uterus to the right. In addition, mild endometriosis and several abdominal adhesions were diagnosed between the intestines, uterus and tumour tissue. Since the patient had also a spontaneous ectopic pregnancy in the right fallopian tube, likely damaging the tissue, the heterotopic ovarian tissue transplantation was the only option to restore her fertility.

**Fig. 2:**
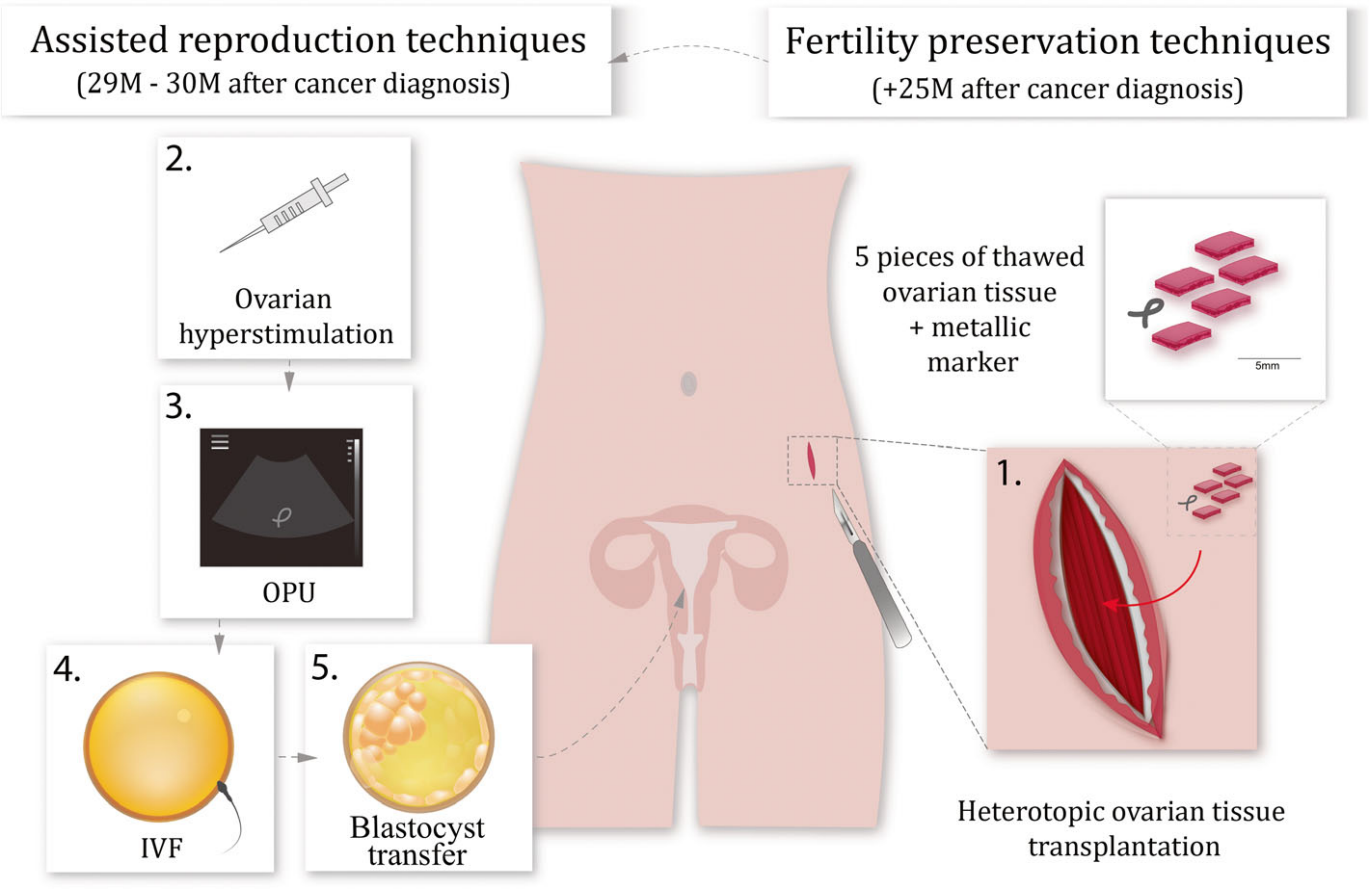
Schematic view of the fertility preservation procedures. Based on the patient’s clinical conditions the heterotopic ovarian tissue transplantation was the only option to restore her fertility after 25 months of cancer diagnosis. (1) 5 pieces of thawed ovarian tissue were transplanted to the submuscular pocket on the left side of the lateral pelvic wall and marked with a metallic marker for better ultrasound visibility. (2) The folliculogenesis was stimulated in the transplanted tissue by using exogenous follicle-stimulating hormone. (3) The oocytes were collected under ultrasound guidance from the grafted tissue (near the metallic marker) only for subsequent (4) IVF and (5) embryo transfer. No follicles were punctured from the ovaries, thus excluding the chance for the spontaneous pregnancy

Thawing of five ovarian cortical pieces was performed using the following steps: the cryovials were placed in the 30 °C water bath until the ice was melted. Then the pieces of cortical strips were incubated in 1 M PrOH and 0.2 M sucrose for 5 min and followed by 0.5 M PrOH and 0.2 M sucrose for 5 min in room temperature. Thereafter the pieces were placed into 0.2 M sucrose in HSA (25 mg/ml) in PBS for 10 min. Heterotopic transplantation was used for thawed pieces of ovarian cortex into a submuscular pocket on the left side of the lateral pelvic wall and marked with a metallic marker for better ultrasound visibility (as shown in Fig. 2).

### Ovarian graft stimulation and fertility treatment

After the ovarian tissue transplantation, during the following three months, the FSH level declined to 14,1 U/L and AMH level raised to 0,3 ng/mL. In the middle of 2013, the patient regained her first spontaneous menstrual cycle after tissue grafting.

For controlled ovarian hyperstimulation, we classified the patient as a possible poor ovarian responder, and used the gonadotropin-releasing hormone (GnRH) agonist long protocol with Diphereline (3.75 mg) administration started on the 21st cycle day. On the third day after the beginning of bleeding, the patient started treatment with exogenous recombinant follicle-stimulating hormone (FSH, Gonal-F, 225 IU per day for 2 days, and 300 IU per day from day 3 to 12). The follicular growth and endometrial thickness were measured using ultrasound combined with serum estradiol (E_2_) measurements to time the injection of human chorionic gonadotropin (hCG) for oocyte meiotic resumption. Recombinant hCG (Ovitrelle, 250 μg) was administered to the patient and the oocyte pickup (OPU) was performed 36 h later.

On the day of OPU, the patient had three follicles of ≥18 mm in the location of the transplanted tissue. Due to the lack of the follicles, the ovaries remained unpunctured and the oocytes were only retrieved from the grafted ovarian tissue, excluding any chance for natural conception. The oocyte retrieval from the transplanted tissue was uncomplicated. All three follicles were aspirated, and two oocytes were obtained. After OPU, the patient was treated with progesterone (Duphaston, 10 mg) by oral administration, until week twelve of gestation. Since the E_2_ level dropped in mid-luteal-phase, the E_2_ (Estrofem, 4 mg orally) was also included in the treatment.

Normal IVF was done with both of the oocytes fertilized and one blastocyst was transferred to the uterus on day 5 after oocyte insemination. A positive serum hCG test (593.1 IU/l) performed two weeks after embryo transfer confirmed pregnancy. Subsequently, clinical pregnancy was ascertained in the sixth week of pregnancy, with the gestational sac measured 2.04 cm, crown-rump length 0.49 cm, and heart beating. The timely development of the pregnancy per IVF embryo transfer precluded the natural conception.

**Fig. 3:**
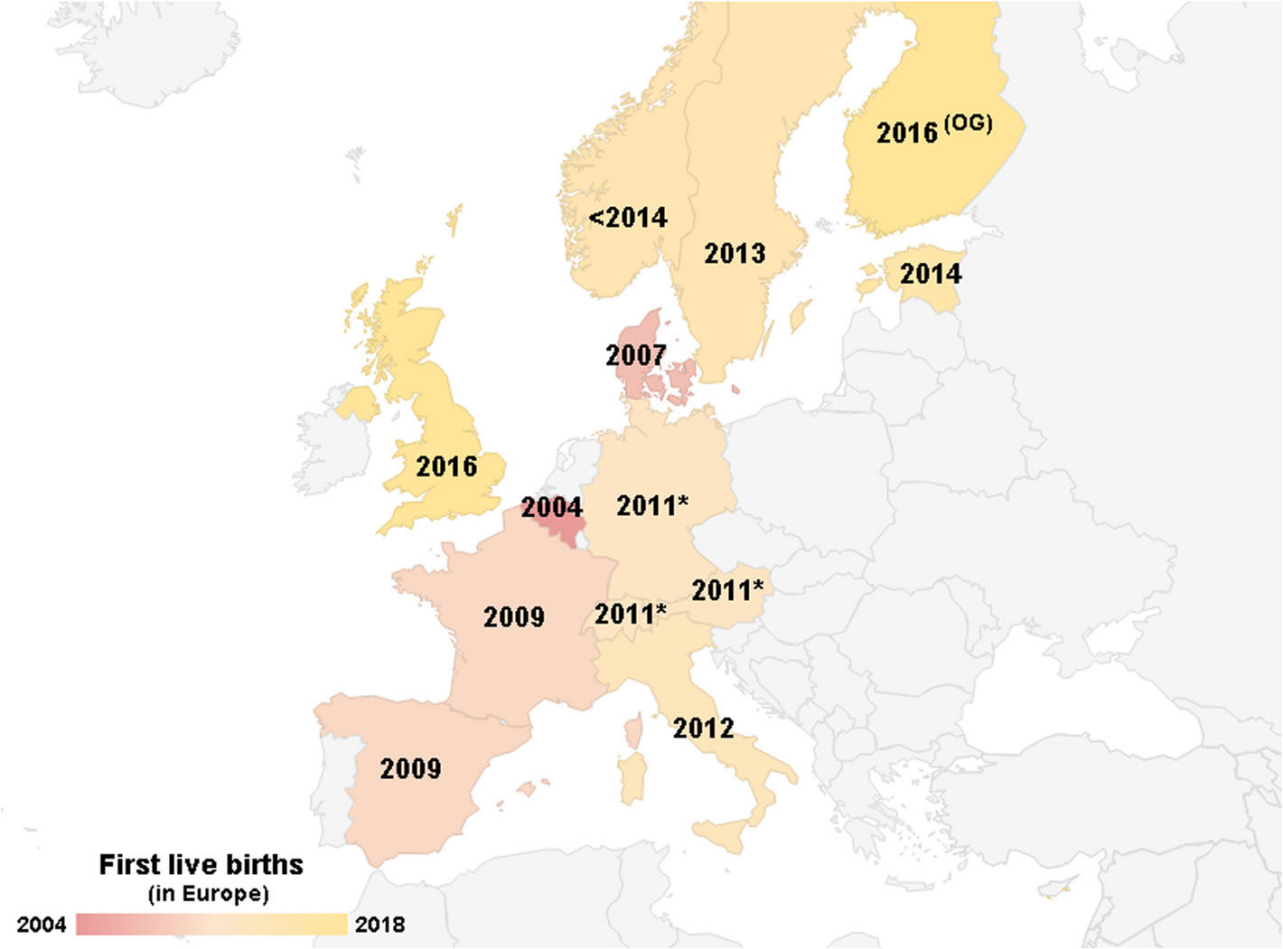
First live births after cryopreserved ovarian tissue transplantation to cancer survivors in European countries. By 2014, live births after cryopreserved ovarian tissue transplantation have been reported in majority of the Western European countries, while Eastern European countries reported this later than Estonia. Abbreviation: *OG* ongoing pregnancy. *FertiProtekt cooperation of ca 100 institutions in Germany, Austria and Switzerland

At week 28 of pregnancy, gestational diabetes was diagnosed, and although the low carbohydrate diet was recommended for the patient, at week 32, additional Metformin (500 mg twice per day) was administered. The healthy boy (3138 g) was born at 39 + 0 weeks of gestation in April 2014 (Figs. 1 and 3).

### The recovery of endogenous ovarian function and follow-up of the child’s health

By the year of 2016, the computed tomography scans had not shown any changes in the breast tissue or formation of metastases. The patient had regained regular 28-day menstrual cycles, for the first time after cancer therapy, indicating the presence of normal ovarian endocrine function. Surprisingly, in 2017, the patient had a spontaneous pregnancy, which could have happened only via recovery of endogenous ovarian function, because the conception via heterotopic transplant would have been possible only using IVF.

At week 20 of the pregnancy, again the gestational diabetes was diagnosed and the patient was recommended low carbohydrate diet. The healthy girl (4100 g) was born at 39 + 0 of gestation in November 2017.

Both of the children have developed normally without major complications. However, both of the children have had some difficulties with the autoimmune diseases such as atopic dermatitis. Also, the first child was diagnosed with juvenile arthritis at the age of 3.5 years.

## Discussion

Current case study reports the first ovarian tissue cryopreservation and heterotopic transplantation for cancer survivor in Estonia and in Eastern Europe. The following successful pregnancy resulted in live birth of a healthy boy in April 2014. Furthermore, years after cancer treatment and ovarian tissue reimplantation, the patient had a spontaneous pregnancy and a healthy girl was born in November 2017, indicating the unexpected recovery of ovarian function.

Ovarian tissue cryopreservation and transplantation are relatively new and promising options to women with cancer diagnosis to preserve their fertility, as the chemotherapy-induced follicular depletion may lead to a loss of menstrual cycle activity that can be transient or permanent. Our patient received adjuvant chemotherapy using six cycles of 5-fluorouracil, epirubicin and cyclophosphamide, followed by the radiation therapy. Cyclophosphamide is a highly gonadotoxic alkylating agent that causes death of primordial and antral follicles leading to ovarian insufficiency [11, 12]. Indeed, the patient developed amenorrhea after the initiation of chemotherapy that evolved to irregular oligomenorrhea after a year. Despite of achieving an ectopic pregnancy, the persistent ovulatory dysfunction was considered as a strong indicator for using cryopreserved ovarian tissue grafting to restore her fertility. Moreover, due to the other reasons for impaired fertility (e.g. cystic benign ovarian tumours, endometriosis and severe pelvic adhesions), the heterotopic ovarian tissue transplantation was conducted two years after the commencement of the cancer therapy.

The folliculogenesis was stimulated in the transplanted tissue and oocytes were collected for IVF and embryo transfer. The healthy boy was born after full-term gestation in 2014, first in Eastern Europe. The possibility of a natural conception after ovarian tissue grafting cannot be entirely excluded, and should, therefore, be also acknowledged. Still, after heterotopic tissue transfer, hormonal stimulation and IVF embryo transfer, as implemented in the current case report, the chance for spontaneous pregnancy did not exist.

Premature ovarian failure after chemotherapy is individual and dependent upon type and dose of the administered cytostatic drug, patients’ age and pretreatment ovarian reserve [8, 13]. Menstrual cycle resumption may occur within the first year after chemotherapy [13, 14]; however, there is always uncertainty over the personalised probability for fertility restoration. A recent study focused on a very early phase of ovarian function recovery for young breast cancer patients, claiming that recovery may follow two different patterns, i.e. demonstrating slow or fast recovery pattern [13]. In women belonging to the slow recovery group, all growing follicles are likely destroyed, possibly due to a higher individual susceptibility to chemotherapy. Conversely, women with a fast recovery may still have some antral follicles left, which resume their growth after the end of treatment [13]. The development of the primordial follicles from the quiescent state to the growing antral follicles takes about six months to accomplish in human ovaries, explaining the relatively slow cycle restoration [13, 15]. In certain patients, the restoration, if any, may take months or even years. Indeed, the patient in the current study received six cycles of cyclophosphamide chemotherapy in 2010–2011 and had a slow and partial recovery to experience irregular oligomenorrhea by the year 2012.

However, by the year 2016 the patient had regained regular 28-day menstrual cycle and had a second child spontaneously conceived in 2017. This is a strong evidence that endogenous ovary had regained its function to produce viable follicles and ovulate mature eggs that are available for fertilization. There may be a possibility that transplanted ovarian tissue could be beneficial to the endogenous ovary. Recent study on mice have shown that grafting from a healthy isogenic ovary to the ovary of chemo-treated host rescued function and fertility of the grafted host ovary and resulted in the production of host-derived offspring [16]. The rescue mechanism is most probably involved with diffusible paracrine substance(s) secreted from the donor ovarian tissue. Signals from transplanted ovarian tissue could prevent apoptosis of granulosa cells in endogenous ovary, promote the formation of new granulosa cells, or affect the vasculature or neuronal circuits in the ovary [16]. Moreover, grafted ovarian tissue may provide a suitable microenvironment to activate oogonial stem cells in postnatal ovary that could differentiate to form new follicles [17, 18]. Still, further elucidation is needed to figure out how grafted ovarian tissue could influence the function of the cells in endogenous ovary.

In our case report, two healthy children were born, one with the aid of heterotopic ovarian tissue transplantation and one through the functional recovery of endogenous ovary. The health and well-being of children conceived after ovarian tissue transplantation remains poorly studied [7, 9, 19]. Thus, every successfully conducted ovarian tissue transplantation procedure and the follow-up information of the child’s health are worth of reporting. Previous studies have demonstrated that children born via ovarian tissue reimplantation have had the average gestational time of 39 + 0 and normal body weight [19]. The child in the current case study was born timely, in 39 + 0 weeks of pregnancy and within the range of internationally accepted normal body weight. The overall health of both children has been good and without major complications. Yet, at the age of 3–4 years, the boy had been diagnosed with juvenile arthritis and both of the children have difficulties with the autoimmune diseases such as atopic dermatitis. However, there is no evidence whatsoever for a causal relationship between these diseases and the mode of conception.

Ovarian tissue cryopreservation and transplantation have been largely experimental and even forbidden in several countries, because of the possible risk for reseeding the cancer cells with the tissue grafting. However, recent meta-analyses studies and large case-series of ovarian tissue transplantations have shown that orthotopic ovarian tissue transplantation is now a valid method which is expanding beyond the experimental stage and already has become an established technique for fertility preservation in multiple centers [19–24]. Yet, there are only two published case reports that describe successful pregnancy and live births after heterotopic ovarian tissue cryopreservation [25, 26], similar to the approach used in the current report.

In addition, there have been no indications of sufficient numbers of malignant cells present in the ovarian tissue to cause recurrence of cancer after ovarian tissue transplantation. Recent analyses have shown that about 7% of patients experience cancer recurrence, but these have not been associated with grafted ovarian tissue [20, 24, 27]. Jadoul et al. also showed that in favour of ovarian tissue cryopreservation, 96% of patients were found to be satisfied with the procedure, which brings a sense of hope to cancer patients to picture themselves in the future [21].

Over 130 live births are reported globally by multicenter and single case reports [6, 9]. However, global access to this procedure remains poor. To our knowledge, most of the successful cases of live births have been reported in western part of Europe, starting in Belgium in 2004 [8] and soon in Denmark in 2007 [28] (Fig. 3). In 2009, the first live births were reported in France and Spain [29, 30]. The cooperation of ca 100 institutions from Germany, Austria and Switzerland at FertiProtekt announced their first live birth after cryopreserved ovarian tissue transplantation in 2011 [31]. Furthermore, there have been live births also in Italy (2012), Sweden (2013), Norway (< 2014), United Kingdom (2016) and also one ongoing pregnancy in Finland [3, 7, 32–35] (Fig. 3). Here we report the first live birth to a cancer survivor patient using previously cryopreserved ovarian tissue in Estonia and also in the eastern part of Europe in 2014 (Fig. 3). Our case study reveals that many more countries have started to acknowledge the importance of fertility counselling, giving the opportunity of motherhood to young female cancer patients.

## Conclusion

Despite that many countries have reported the first implementation of the ovarian tissue freezing and transplantation protocols, the data is still limited on the effectiveness of heterotopic ovarian transplant techniques and more long-term follow-up studies and case reports are needed to describe ovarian function recovery and the health of children born. However, the information gathered from large national and international multicentre studies, and clinical case reports from individual patients is very encouraging that ovarian tissue freezing and transplantation have completed an important experimental phase and are ready for wider clinical use in female fertility preservation.

## Abbreviations

E_2_: estradiol
GnRH: gonadotropin-releasing hormone
hCG: human chorionic gonadotropin
HSA: human serum albumin
IVF: in vitro fertilisation
OPU: ovum pickup
PrOH: 1,2-propanediol
